# Sex-Based Differences in Psychosocial Recovery Following Posterior Spinal Fusion for Adolescent Idiopathic Scoliosis

**DOI:** 10.3390/healthcare14040534

**Published:** 2026-02-21

**Authors:** Evren Karaali, Osman Çiloğlu, Burak Keklikçioğlu, Oğuzhan Çiçek, Hüseyin Mehmet Gürbüz, Asiye Arıcı Gürbüz, Mustafa Turan Yakar

**Affiliations:** 1 Department of Orthopedics and Traumatology, Adana City Training and Research Hospital, University of Health Sciences, Adana 01230, Turkey; 2 Department of Child and Adolescent Psychiatry, Adana City Training and Research Hospital, University of Health Sciences, Adana 01230, Turkey

**Keywords:** adolescent idiopathic scoliosis, spinal fusion, health-related quality of life, sex characteristics

## Abstract

**Background:** Although posterior spinal fusion (PSF) for adolescent idiopathic scoliosis (AIS) reliably improves radiographic alignment, radiological correction alone does not necessarily reflect postoperative recovery, particularly in terms of psychosocial well-being. Patient-reported outcome measures (PROMs) have become central to outcome assessment in AIS; however, the relative contributions of disease-specific and generic instruments, sex-based differences, and functional recovery, including return to sports, remain unclear. **Methods:** This prospective single-center cohort study encompassed adolescents aged 13–18 years who underwent PSF for AIS between December 2020 and November 2023. All included patients had a minimum postoperative follow-up of 24 months at the time of analysis. Health-related quality of life (HRQoL) was evaluated preoperatively and at least 2 years postoperatively using the Scoliosis Research Society–22 revised questionnaire (SRS-22r) and the Pediatric Quality of Life Inventory (PedsQL) Teen Report (ages 13–18 years). Outcomes were compared between male and female patients. Return to the preoperative level of sports was evaluated as a functional outcome. **Results:** Overall, 108 adolescents with AIS (32 males, 76 females) were included. Male patients were slightly older at the time of surgery, whereas baseline radiographic characteristics, treatment patterns, and follow-up duration were comparable between sexes. At preoperative assessment, male patients reported higher SRS-22r self-image and mental health scores compared with female patients (both *p* < 0.001). These differences were no longer present at the last follow-up (all *p* > 0.05). Emotional functioning improved significantly in both sexes (*p* < 0.001), whereas changes in pain and physical function were limited. The total PedsQL score increased significantly in female patients but not in males (*p* = 0.521). Patients across all Lenke curve types demonstrated postoperative improvements. Those with Lenke type 1 exhibited higher mean changes in SRS-22r and PedsQL total scores; however, differences in change scores between Lenke types demonstrated no statistical significance. At final follow-up, 93.5% of patients had not returned to their preoperative level of physical activity. **Conclusions:** PSF for AIS was associated with domain-specific improvements in HRQoL, predominantly reflecting psychosocial domains rather than changes in pain or physical function. Preoperative sex-based differences resolve postoperatively, and patients with Lenke curve types demonstrate improvements, with a tendency toward greater gains in Lenke type 1. Despite these improvements, return to preoperative sport levels remains restricted, indicating a gap between patient-reported recovery and functional reintegration.

## 1. Introduction

Adolescent idiopathic scoliosis (AIS) is a three-dimensional spinal deformity of unknown etiology developing between ages 10–18 years and is the most common form of idiopathic scoliosis in adolescents. Posterior spinal fusion (PSF) with instrumentation has been considered the standard surgical treatment for moderate to severe AIS, aiming to correct the deformity and stabilize the spine. PSF enhances radiographic alignment and limits curve progression; however, radiological correction alone does not sufficiently reflect postoperative recovery in adolescents, particularly in terms of daily function and psychosocial well-being. Accordingly, current outcome assessment in AIS prioritizes validated patient-reported outcome measures (PROMs) along with radiographic parameters [1,2].

The Scoliosis Research Society–22 revised questionnaire (SRS-22r) is the most frequently employed disease-specific PROM in AIS and has been demonstrated to be responsive to postoperative change, particularly in domains of significance to adolescents, including self-image and mental health [3,4]. In comparison, the Pediatric Quality of Life Inventory (PedsQL) Teen Report (ages 13–18 years) is a validated generic health-related quality-of-life (HRQoL) instrument that assesses broader aspects of physical, emotional, social, and school functioning [5,6]. Considering the various constructs targeted by disease-specific and generic measures, variations in domain sensitivity following AIS surgery are anticipated and do not indicate inconsistency; rather, the combined use of both instruments enables a more comprehensive and clinically meaningful recovery assessment [7].

Sex-based differences in HRQoL remain a clinically relevant but not a well-elucidated aspect of AIS outcomes. Female patients frequently verbalize poorer preoperative psychosocial status, particularly in appearance-related domains, despite similar clinical severity, and previous studies have suggested that these differences can diminish postoperatively [8,9]. Besides changes in patient-reported outcomes, return to preoperative sport and physical activity levels has become a crucial functional outcome for adolescents; however, it is inconsistently reported and not routinely incorporated into HRQoL-based assessments [10]. A clearer understanding of how sex-based HRQoL differences change over time and how PROM improvements affect return to sports may help refine postoperative counseling and more effectively manage patient expectations.

This study aimed to assess sex- and time-based changes in HRQoL following PSF for AIS using a disease-specific instrument (SRS-22r) and a generic instrument (PedsQL Teen Report, ages 13–18 years) and evaluate return to the preoperative sport level as a functional outcome alongside PROMs. We hypothesized that female patients would report worse preoperative self-image and mental health scores than male patients despite comparable clinical and radiographic characteristics; that sex-based differences would decline by the last follow-up, with both groups demonstrating improvements; and that gains in patient-reported outcomes would not necessarily correspond to full return to the preoperative sport level, reflecting the distinction between psychosocial recovery and high-demand functional reintegration after fusion surgery. However, despite increasing emphasis on patient-reported outcomes in AIS surgery, limited evidence exists regarding how sex-based psychosocial recovery patterns relate to functional reintegration, particularly return to preoperative activity level. Furthermore, the interaction between curve type, sex, and multidimensional HRQoL recovery remains incompletely characterized.

## 2. Methods

### 2.1. Study Design and Patient Selection

This prospective single-center study encompassed adolescents who underwent PSF for AIS from December 2020 to November 2023 at Adana City Hospital. Follow-up data were collected through November 2025 to ensure that all included patients met the predefined minimum 2-year postoperative follow-up criterion. The local ethics committee provided ethical approval, and written informed consent was obtained from the parents/legal guardians, with assent obtained from the participants when appropriate. Inclusion criteria were patients aged 13–18 years diagnosed with AIS who underwent PSF and had complete preoperative and postoperative PROMs with a minimum follow-up of 2 years. Exclusion criteria were patients with non-idiopathic, neuromuscular, or syndromic scoliosis; a history of previous spinal surgery; or incomplete follow-up data.

### 2.2. Surgical Procedure

All patients underwent posterior spinal fusion with segmental pedicle screw instrumentation using a posterior-only approach. Curve correction was achieved through rod derotation and standard deformity correction maneuvers. The selection of upper and lower instrumented vertebrae was based on Lenke classification and sagittal balance parameters. Intraoperative neuromonitoring was routinely used. No anterior procedures were performed in this cohort.

### 2.3. Postoperative Management and Rehabilitation

All patients followed a standardized postoperative care protocol. Early mobilization was initiated on the first postoperative day under supervision. A thoracolumbosacral orthosis (TLSO) brace was routinely prescribed for approximately 45 days following surgery. The brace was intended to provide temporary external support during the early postoperative phase, facilitating patient confidence during mobilization and daily activities rather than serving as a primary stabilizing measure.

All patients received standardized postoperative rehabilitation instructions, including supervised in-hospital mobilization and structured home-based exercise recommendations after discharge. No patient-specific or sex-based differences in rehabilitation protocols were applied.

Patients were encouraged to resume daily activities gradually, with avoidance of high-impact sports during the early recovery period. Return to non-contact sports was generally permitted after radiographic evidence of progressive fusion, whereas unrestricted activity was allowed following clinical and radiographic confirmation of stable fusion. Final decisions regarding return to sport were individualized based on clinical evaluation and radiographic findings. Postoperative care and rehabilitation guidance were consistent across cohorts.

### 2.4. Follow-Up Protocol

Patients were followed postoperatively according to a standardized clinical protocol. Routine follow-up visits were scheduled at approximately 3 weeks, 6 weeks, 3 months, 6 months, and 12 months after surgery, and annually thereafter. Outcome measures for the purpose of this study were collected at the final follow-up visit, with a minimum follow-up duration of 24 months.

### 2.5. Outcome Measures

Preoperative assessments were conducted at the time of hospital admission prior to surgery. Postoperative outcome measures were collected at the final follow-up visit, which occurred at approximately 24 months postoperatively, with all patients having a minimum follow-up duration of 24 months.

### 2.6. SRS-22r

The SRS-22r, a validated disease-specific instrument, was used for evaluating HRQoL. Domain scores (pain, function, self-image, mental health, and satisfaction) were calculated as the mean of their corresponding items, with higher scores indicating better HRQoL. The satisfaction domain (items 21–22) was analyzed only at the last follow-up as it reflects postoperative treatment appraisal and is not applicable preoperatively [11]. For valid preoperative–postoperative comparisons, subtotal scores based on the first 20 items were used in time-based analyses [12].

### 2.7. PedsQL Teen Report (Ages 13–18 Years)

The PedsQL Teen Report (ages 13–18 years), a validated generic instrument that evaluates physical, emotional, social, and school functioning, was employed for assessing generic HRQoL. Scoring followed the standard PedsQL scoring algorithm and interpretation guidance, with higher scores indicating better HRQoL [13]. To capture aspects of general functioning and participation in patients with AIS, the PedsQL was used alongside the disease-specific SRS-22r. The SRS-22r and PedsQL Teen Report (ages 13–18 years) were selected to facilitate age-appropriate assessment and offer complementary information on scoliosis-specific psychosocial concerns and overall health-related quality of life in adolescents.

Validated Turkish versions of both the SRS-22r and the PedsQL Teen Report were used in this study, and prior studies have demonstrated their reliability and validity in Turkish adolescent populations [11,13].

### 2.8. Functional Outcome: Return to Sports

Return to the preoperative sport level was recorded as a binary variable (yes/no) at the last follow-up. Return to sport was defined as return to the patient’s own preoperative level of recreational or organized physical activity, irrespective of sport type, as all patients reported engagement in physical activity before surgery. This outcome was included because return to sports may occur later than improvements in PROMs and has become a crucial focus in postoperative AIS recovery studies [14,15]. Preoperative sports participation was assessed in terms of self-reported engagement in physical activity; however, detailed characterization of sport type, frequency, or competitive level was not systematically recorded.

### 2.9. Radiographic Assessment

Standard standing posteroanterior and lateral radiographs were obtained preoperatively and at follow-up. The primary curve magnitude was measured using the Cobb method. Curve patterns were classified according to the Lenke classification system. Radiographic parameters were used to describe baseline deformity characteristics and were not considered primary outcome measures in this study.

### 2.10. Statistical Analysis

Patients were stratified by sex (males vs. females). Continuous variables were summarized as means ± standard deviations and categorical variables as counts and percentages. Statistical analyses were performed using IBM SPSS Statistics for Windows, Version 26.0 (IBM Corp., Armonk, NY, USA). Normality of continuous variables was assessed using the Shapiro–Wilk test. Between-group comparisons employed the Mann–Whitney U tests and chi-square tests for continuous and categorical data, respectively. Within-group preoperative–postoperative comparisons were evaluated using Wilcoxon signed-rank tests as appropriate. Expanded analyses were performed for assessing sex differences at each time point and within-sex changes across time. Comparisons across Lenke curve types were conducted using the Kruskal–Wallis test due to unequal subgroup sizes. A *p*-value of <0.05 was considered statistically significant. Given the hypothesis-driven and domain-specific nature of the primary analyses, formal adjustment for multiple comparisons was not applied. Lenke subgroup analyses were performed on an exploratory basis due to unequal and limited subgroup sizes. Clinical relevance of SRS-22r changes was interpreted in relation to previously published minimum clinically important difference (MCID) thresholds [4].

## 3. Results

A total of 129 patients underwent posterior spinal fusion for AIS during the study period. Twenty-one patients were excluded from the final analysis. Exclusion reasons included screw misplacement (*n* = 1), proximal junctional kyphosis (*n* = 2), pseudarthrosis (*n* = 1), and adding-on phenomenon (*n* = 2). In addition, cases in which Ponte osteotomies were performed (*n* = 3) were excluded to ensure procedural homogeneity and minimize potential variability in postoperative pain and functional assessment related to more extensive posterior column release. Furthermore, twelve patients were excluded due to incomplete follow-up or missing PROM data. The final study population consisted of 108 patients who met all inclusion criteria. Overall, 108 patients were included, comprising 32 males (29.6%) and 76 females (70.4%). At the time of surgery, male patients were slightly older than female patients (14.59 ± 0.91 vs. 14.18 ± 0.78 years, *p* = 0.029). Preoperative Cobb angle, Cobb angle at the last follow-up, and follow-up duration were comparable between sexes (all *p* > 0.05). The distribution of Lenke curve types did not vary between male and female patients (*p* = 0.923). Furthermore, rates of prior brace treatment and exercise therapy were comparable between the groups. At the last follow-up, most patients (*n* = 101, 93.5%) had not returned to their own preoperative level of physical activity, with both sexes exhibiting no differences (*p* > 0.05) (Table 1).

At the preoperative assessment, male patients reported higher self-image, mental health, and SRS-22r subtotal (20-item) scores than female patients (all *p* < 0.001). Preoperative pain or function scores showed no sex-based differences. At the last follow-up, previously observed preoperative sex-based differences in psychosocial domains were no longer statistically significant, and male and female patients exhibited comparable scores across all SRS-22r domains, including pain, function, self-image, mental health, subtotal, and satisfaction (all *p* > 0.05). Within-group analyses revealed postoperative improvements from baseline to the last follow-up in both sexes. In male patients, self-image, mental health, and SRS-22r subtotal scores showed improvements (all *p* < 0.001). In female patients, improvements in self-image and mental health scores were noted (all *p* < 0.001). Both groups demonstrated minimal changes in pain and function scores. Postoperative SRS-22r satisfaction scores, assessed only at the last follow-up, were comparable between sexes (*p* > 0.05) (Table 2).

PedsQL Teen Report scores were analyzed on the standard 0–100 transformed scale, with higher values indicating better health-related quality of life. Preoperatively, male patients reported higher PedsQL scores than female patients across physical, emotional, social, school, and total domains. At the last follow-up, no sex-based differences were noted in any PedsQL domain or in the total score (all *p* > 0.05). Within-group analyses revealed domain-specific patterns of change. In both sexes, emotional and school functioning scores increased significantly postoperatively (all *p* < 0.001), whereas physical functioning scores decreased and social functioning scores showed modest declines. The total PedsQL score increased significantly in female patients, while no statistically significant change was observed in male total scores (*p* = 0.521) (Table 3).

Both the SRS-22r and PedsQL Teen Report showed postoperative improvement and resolution of preoperative sex-based differences, with both instruments exhibiting comparable patterns.

Postoperative changes in SRS-22r and PedsQL Teen (ages 13–18 years) scores were observed across all patients with Lenke curve types. Given the small sample sizes in certain Lenke subgroups, these findings should be interpreted cautiously. The magnitude of change varied by curve classification. Patients with Lenke type 1 exhibited the highest mean increase in both PedsQL (3.35 ± 6.02) and SRS-22r (0.70 ± 0.18) total scores. Moreover, those with Lenke type 1 demonstrated higher mean changes in several SRS-22r domains, including function, pain, self-image, and mental health, than other Lenke groups. Certain domains within other subtypes, such as PedsQL emotional scores in Lenke type 2 and selected SRS-22r domains in Lenke type 6, exhibited numerically higher changes. However, between-group comparisons revealed no statistically significant differences in change scores across Lenke classifications for either SRS-22r or PedsQL domains (all *p* > 0.05, Kruskal–Wallis test) (Table 4).

## 4. Discussion

Postoperative recovery in adolescents undergoing PSF for idiopathic scoliosis was assessed using PROMs and a functional endpoint. Although radiographic correction represents a significant surgical goal, our findings suggest that postoperative recovery is primarily perceived in psychosocial areas rather than in pain or physical function. Clinically, this finding is anticipated as several adolescents proceed with surgery owing to appearance and self-image-related concerns rather than functional limitations. Therefore, patient-centered outcome assessment is particularly crucial in this population, where psychological well-being plays a central role in the overall quality of life.

Several adolescents, particularly female patients, applied for surgery, reporting preserved pain and functional status but marked impairment in self-image and mental health. This finding reflects a common clinical pattern in AIS, wherein patients remain physically capable but experience significant appearance and social perception-associated psychological distress. In this context, scoliosis should be viewed as a structural deformity and a condition with significant psychosocial impact during adolescence. The observed preoperative sex-based differences, with female patients reporting worse self-image and mental health despite comparable radiographic severity, align with previous studies underscoring greater appearance-related concerns among adolescent girls with scoliosis [8,16].

At the last follow-up, these preoperative sex-based differences in psychosocial domains were no longer statistically significant, reflecting convergence between groups rather than persistent between-sex disparities. Similar patterns have been previously reported and may relate to changes in trunk contour and external appearance following surgical correction [17,18]. This convergence occurred without sex-based differences in postoperative pain or functional scores, indicating that psychosocial recovery may evolve independently of measurable changes in physical function. Overall, the present findings demonstrate an association between surgical correction and improved psychosocial well-being, while causal mechanisms cannot be determined from these data.

Postoperatively, patients mainly exhibited improvements in psychosocial areas, particularly self-image, mental health, and overall quality of life. By contrast, pain and changes in physical function were minimal, aligning with the relatively preserved baseline functional status of this cohort. Although a statistically significant decrease in the SRS-22r pain score was observed in male patients, the absolute change was small (0.17 points) and below the commonly reported minimum clinically important difference for the pain domain [4]. Therefore, this finding is unlikely to represent clinically meaningful deterioration. These findings suggest that for several adolescents, the primary benefit of surgery is not restoring physical capacity but rather improving the association between physical appearance and self-perception. These types of changes can impact aspects, including confidence, emotional well-being, and social participation, which are frequently inadequately addressed by standard clinical measures. This interpretation agrees with content validity and concept elicitation studies showing that appearance-related concerns and self-perception are key issues for adolescents with AIS [9,19].

The combined use of the SRS-22r and PedsQL provided complementary insight into postoperative recovery patterns. The disease-specific SRS-22r appeared more sensitive to scoliosis-related psychosocial changes, particularly in self-image and mental health, whereas the generic PedsQL reflected broader domain-specific trends over time. Emotional and school functioning scores increased significantly following surgery, while physical functioning scores decreased and social functioning showed modest declines. Importantly, total PedsQL scores improved significantly in female patients but did not change significantly in male patients [5,7,20]. These results support the combined use of disease-specific and generic PROMs for providing a more clinically interpretable view of postoperative recovery. In addition to the SRS-22r, other SRS-based instruments such as the SRS-30 have been used in clinical practice. The SRS-30 includes additional postoperative-specific items that may provide more detailed insight into treatment appraisal and recovery after surgery. Recent literature has highlighted the potential clinical value of incorporating broader SRS-based measures when evaluating outcomes in AIS patients. Nevertheless, given the extensive validation, widespread adoption, and comparability of the SRS-22r in longitudinal AIS research, we considered it an appropriate and reliable instrument for outcome assessment in this cohort [21]. Importantly, the magnitude of improvement observed in SRS-22r self-image and mental health domains exceeded commonly cited MCID thresholds, supporting clinical relevance beyond statistical significance [4]. In contrast, changes in pain and function were small in magnitude in this cohort with relatively preserved baseline physical status.

Another observation is associated with curve classification. Although all patients with Lenke subtypes exhibited postoperative improvements, those with Lenke type 1 curves tended to show greater gains in both SRS-22r and PedsQL total scores. This numerical tendency may relate to differences in curve pattern and correction characteristics; however, causal inferences regarding the mechanisms underlying curve-specific recovery cannot be drawn from the present data. More complex deformity patterns may require longer fusion constructs and could influence patient-perceived recovery despite satisfactory radiographic correction. However, no statistically significant differences were observed between Lenke classifications, suggesting that meaningful patient-reported improvement can be achieved across curve types. Although patients with Lenke type 1 curves demonstrated numerically greater improvements, subgroup sizes were uneven and limited in certain classifications. Therefore, these findings should be interpreted as exploratory and hypothesis-generating rather than definitive evidence of curve pattern-specific differences.

A gap remains between perceived recovery and functional reintegration despite developments in psychosocial areas. Most patients were unable to return to their preoperative sport level, even during the long-term follow-up. Similar findings have been reported following AIS fusion, with delayed or incomplete return to sports despite promising patient-reported outcomes [14,15,22]. Residual spinal stiffness, altered biomechanics, concern about reinjury, and conservative activity recommendations are potential contributors [23,24]. These findings suggest that improvements in the quality of life, particularly in relation to athletic participation, do not necessarily correspond to complete functional recovery. The low rate of return to the preoperative sport level (6%) should be interpreted cautiously, as multiple factors beyond structural correction may influence postoperative activity patterns, including patient preference, confidence, rehabilitation strategies, and surgeon guidance. Posterior spinal fusion alters spinal mobility characteristics; however, the extent to which this directly limits high-demand athletic participation cannot be determined from the present study. In addition, adolescents and their families may adopt protective or avoidance behaviors after surgery, particularly in the setting of instrumented fusion. Therefore, although psychosocial domains such as self-image and mental well-being improved substantially, these gains do not necessarily equate to full restoration of preoperative physical activity patterns. These findings underscore that psychosocial recovery and high-demand functional reintegration represent distinct dimensions of postoperative outcome in AIS surgery. While patients may experience substantial improvement in self-image and emotional well-being, restoration of preoperative activity intensity appears to follow a different and more limited trajectory. Reported return-to-sport rates after AIS fusion vary widely in the literature, ranging from partial return to delayed reintegration depending on sport intensity, fusion length, and follow-up duration [14,15,22]. Our findings fall within the lower spectrum of reported rates and should be interpreted within this broader clinical context. These heterogeneous findings across studies underscore the complexity of postoperative athletic reintegration in AIS and suggest that functional recovery cannot be attributed solely to radiographic correction. Some studies have demonstrated earlier return in low-impact activities but more cautious progression in high-demand sports. Our findings are consistent with this variability and support the concept that psychosocial recovery may precede complete athletic reintegration. Importantly, return-to-sport outcomes may be influenced by multiple interrelated factors beyond structural correction alone, including surgeon recommendations for gradual reintegration, patient or family preference, rehabilitation strategies, and psychological factors such as fear-avoidance or kinesiophobia. The present study did not systematically assess these variables; therefore, causal inferences regarding biomechanical limitation cannot be drawn from these data.

These findings indicate that pain and function scores alone cannot adequately assess outcomes of AIS surgery. In adolescents with relatively preserved baseline physical capacity, postoperative benefit appears to be more prominently reflected in psychosocial domains than in measurable changes in physical function. Recognizing this distinction may guide patient selection, enhance preoperative counseling, and support postoperative strategies that address physical and psychological recovery. Given the observational design, the number of domain-specific comparisons performed, and the exploratory nature of subgroup analyses, these findings should be interpreted with appropriate caution.

The strongest aspect of our study is its prospective design with standardized outcome assessment and a minimum 2-year follow-up. However, the overall sample size was modest, and subgroup analyses according to the Lenke classification were limited by unequal and small subgroup sizes. Therefore, observed trends favoring Lenke type 1 curves should be interpreted with caution and considered hypothesis-generating rather than definitive. Furthermore, PROMs were subjective and might be influenced by psychosocial factors that were indirectly assessed. Return to sports was assessed as a binary outcome without detailed information regarding activity type, competitive level, timing of return, or psychosocial factors such as fear-avoidance, which may limit the interpretation of functional reintegration. Finally, the unequal sex distribution reflects the epidemiological predominance of AIS in females; however, this imbalance may limit statistical power for detecting subtle sex-based differences.

## 5. Conclusions

Our findings indicate that PSF for AIS is associated with domain-specific improvements in HRQoL, mainly associated with the psychosocial domain, including self-image and mental well-being, while changes in pain and physical functioning were limited. Preoperative sex-based differences resolve postoperatively, indicating comparable psychological recovery between male and female patients. Although patients with Lenke curve types demonstrated improvements, those with Lenke type 1 curves tended to exhibit greater numerical gains, although no statistically significant differences were observed between curve classifications, and any apparent variation in improvement across curve types should be interpreted cautiously. Despite these improvements, most adolescents were unable to return to their preoperative sport level, indicating a disparity between patient-reported improvement and functional reintegration. These findings suggest that psychosocial recovery may precede full physical reintegration after AIS surgery. Longer-term follow-up is warranted to determine whether functional outcomes, including return to sport, continue to evolve over time. Overall, these findings suggest that in adolescents with AIS and preserved baseline physical function, the most pronounced postoperative changes occur in psychosocial domains rather than in measurable physical performance. Future prospective studies with larger and more balanced cohorts are needed to validate these findings and to better delineate the interplay between psychosocial recovery and functional reintegration following AIS surgery.

## Figures and Tables

**Table 1:** Demographic and clinical characteristics by sex.

	Male (*n* = 32)	Female (*n* = 76)	*p* Value
Age at surgery (years)	14.59 ± 0.91	14.18 ± 0.78	0.029
Preoperative Cobb angle (°)	54.34 ± 5.46	52.75 ± 4.76	0.177
Cobb angle at last follow-up (°)	0.97 ± 1.71	1.13 ± 1.63	0.406
Follow-up duration (months)	25.88 ± 1.01	26.21 ± 1.24	0.145
Brace treatment, *n* (%)	19 (59.4%)	48 (63.2%)	0.879
Exercise treatment, *n* (%)	20 (62.5%)	46 (60.5%)	1.000
Return to preoperative sport level, *n* (%)	2 (6.2%)	5 (6.6%)	1.000

Note: Brace treatment and exercise therapy refer to preoperative conservative management.

**Table 2:** Sex- and time-based comparison of SRS-22r outcomes.

	Male Preop	Female Preop	p^1^	Male Last FU	Female Last FU	p^2^	p^3^ (Male)	p^4^ (Female)
Pain	4.91 ± 0.19	4.88 ± 0.21	0.259	4.74 ± 0.35	4.63 ± 0.38	0.106	0.005	<0.001
Function	4.47 ± 0.24	4.43 ± 0.26	0.641	4.38 ± 0.13	4.39 ± 0.16	0.617	0.135	0.182
Self-image	3.33 ± 0.23	2.48 ± 0.28	<0.001	4.78 ± 0.28	4.84 ± 0.27	0.214	<0.001	<0.001
Mental health	4.26 ± 0.27	3.98 ± 0.32	<0.001	4.81 ± 0.33	4.65 ± 0.45	0.221	<0.001	<0.001
Subtotal (20 items)	4.24 ± 0.13	3.94 ± 0.15	<0.001	4.68 ± 0.14	4.63 ± 0.18	0.222	<0.001	<0.001
Satisfaction †				4.91 ± 0.24	4.90 ± 0.26	0.922		

p^1^: Male vs. Female at preoperative evaluation. p^2^: Male vs. Female at last follow-up. p^3^: Preoperative vs. last follow-up within male group. p^4^: Preoperative vs. last follow-up within female group. † The satisfaction domain of the SRS-22r (questions 21–22) reflects postoperative treatment appraisal and is therefore reported only at last follow-up. FU: follow-up.

**Table 3:** Sex- and time-based comparison of PedsQL Teen Report (ages 13–18) outcomes (0–100 scale).

Domain	Male Preop	Female Preop	p^1^	Male Last FU	Female Last FU	p^2^	p^3^ (Male)	p^4^ (Female)
Physical	96.00 ± 4.75	92.75 ± 6.75	0.006	81.00 ± 11.25	80.75 ± 11.25	0.900	<0.001	<0.001
Emotional	89.25 ± 6.00	80.75 ± 11.50	<0.001	94.75 ± 6.50	94.25 ± 5.75	0.625	<0.001	<0.001
Social	96.50 ± 5.25	93.50 ± 6.25	0.013	91.50 ± 5.25	90.75 ± 5.25	0.545	<0.001	0.002
School	61.50 ± 11.50	68.50 ± 12.50	0.020	88.25 ± 8.00	88.50 ± 7.75	0.687	<0.001	<0.001
Total	87.25 ± 3.50	85.00 ± 5.50	0.030	87.75 ± 6.00	87.50 ± 5.00	0.767	0.521	<0.001

p^1^: Male vs. Female at preoperative evaluation. p^2^: Male vs. Female at last follow-up. p^3^: Preoperative vs. last follow-up within male group. p^4^: Preoperative vs. last follow-up within female group. All scores are reverse-scored and linearly transformed to a 0–100 scale; higher scores indicate better health-related quality of life. FU: follow-up.

**Table 4:** Changes in SRS-22r and PedsQL Scores by Lenke Classification.

Lenke Type (*n*)	PedsQL Total Δ	Physical Δ	Emotional Δ	Social Δ	School Δ	SRS Total Δ	Function Δ	Pain Δ	Self-Image Δ	Mental Health Δ
Type 1 (*n* = 49)	3.35 ± 6.02	−10.78 ± 11.01	11.74 ± 11.16	−2.14 ± 6.46	23.06 ± 15.44	0.70 ± 0.18	0.74 ± 0.21	0.80 ± 0.19	0.73 ± 0.20	0.79 ± 0.18
Type 2 (*n* = 15)	1.52 ± 6.87	−12.92 ± 9.51	15.00 ± 12.96	−4.00 ± 7.12	16.67 ± 17.18	0.62 ± 0.22	0.69 ± 0.26	0.72 ± 0.24	0.65 ± 0.23	0.70 ± 0.21
Type 3 (*n* = 8)	0.14 ± 5.28	−17.97 ± 9.26	7.50 ± 7.56	−2.50 ± 7.56	24.38 ± 10.84	0.55 ± 0.22	0.61 ± 0.25	0.63 ± 0.27	0.58 ± 0.24	0.66 ± 0.22
Type 4 (*n* = 4)	0.82 ± 5.78	−12.50 ± 13.26	13.75 ± 10.31	−7.50 ± 8.66	17.50 ± 11.90	0.59 ± 0.09	0.68 ± 0.10	0.78 ± 0.08	0.60 ± 0.09	0.72 ± 0.07
Type 5 (*n* = 27)	0.48 ± 5.79	−15.51 ± 15.42	9.26 ± 7.03	−4.26 ± 6.00	22.04 ± 17.45	0.57 ± 0.21	0.64 ± 0.24	0.69 ± 0.23	0.62 ± 0.22	0.68 ± 0.20
Type 6 (*n* = 5)	0.87 ± 3.87	−13.75 ± 5.68	7.00 ± 8.37	−5.00 ± 7.07	20.00 ± 14.14	0.84 ± 0.39	0.88 ± 0.41	0.91 ± 0.36	0.86 ± 0.38	0.90 ± 0.35
*p* value	0.348	0.627	0.426	0.304	0.681	0.169	0.057	0.240	0.142	0.146

Values are presented as mean ± standard deviation. Δ indicates change from preoperative assessment to last follow-up (postoperative minus preoperative). Between-group comparisons were performed using the Kruskal–Wallis test. PedsQL scores are reverse-scored and linearly transformed to a 0–100 scale; higher scores indicate better health-related quality of life.

## Data Availability

The data supporting the findings of this study are not available duo to patient privacy and ethical restrictions but are available from the corresponding author upon reasonable request.
